# Patient-derived orthotopic xenograft models for cancer of unknown primary precisely distinguish chemotherapy, and tumor-targeting *S. typhimurium* A1-R is superior to first-line chemotherapy

**DOI:** 10.1038/s41392-018-0016-7

**Published:** 2018-04-27

**Authors:** Kentaro Miyake, Tasuku Kiyuna, Masuyo Miyake, Kei Kawaguchi, Sang Nam Yoon, Zhiying Zhang, Kentaro Igarashi, Sahar Razmjooei, Sintawat Wangsiricharoen, Takashi Murakami, Yunfeng Li, Scott D. Nelson, Tara A. Russell, Arun S. Singh, Yukihiko Hiroshima, Masashi Momiyama, Ryusei Matsuyama, Takashi Chishima, Shree Ram Singh, Itaru Endo, Fritz C. Eilber, Robert M. Hoffman

**Affiliations:** 1 AntiCancer, Inc., San Diego, CA USA; 2 Department of Surgery, University of California, San Diego, CA USA; 3 Department of Gastroenterological Surgery, Yokohama City University Graduate School of Medicine, Yokohama, Japan; 4 Department of Pathology, University of California, Los Angeles, CA USA; 5 Division of Surgical Oncology, University of California, Los Angeles, CA USA; 6 Division of Hematology-Oncology, University of California, Los Angeles, CA USA; 7 Basic Research Laboratory, National Cancer Institute, Frederick, MD USA

## Abstract

Cancer of unknown primary (CUP) is a recalcitrant disease with poor prognosis because it lacks standard first-line therapy. CUP consists of diverse malignancy groups, making personalized precision therapy essential. The present study aimed to identify an effective therapy for a CUP patient using a patient-derived orthotopic xenograft (PDOX) model. This paper reports the usefulness of the PDOX model to precisely identify effective and ineffective chemotherapy and to compare the efficacy of *S. typhimurium* A1-R with first-line chemotherapy using the CUP PDOX model. The present study is the first to use a CUP PDOX model, which was able to precisely distinguish the chemotherapeutic course. We found that a carboplatinum (CAR)-based regimen was effective for this CUP patient. We also demonstrated that *S. typhimurium* A1-R was more effective against the CUP tumor than first-line chemotherapy. Our results indicate that *S. typhimurium* A1-R has clinical potential for CUP, a resistant disease that requires effective therapy.

## Cancer therapeutics: Mouse model reveals best treatments for resistant cancer

The genetically-engineered strain of Salmonella A1-R effectively targets human metastatic cancers of unknown primary (CUP) in a mouse model. In up to 5% of advanced cancer diagnosis the primary site of cancer growth cannot be identified. In these cases, prognosis is poor as the most appropriate therapies are determined by the cancer’s tissue of origin. A study led by US researchers Shree Ram Singh, Fritz C. Eilber and Robert M. Hoffman examined the effect of four standard chemotherapeutic drugs and Salmonella A1-R on CUP progression in mice implanted with a lymph node tumor resected from a patient’s neck. To mimic the tumor microenvironment in the patient, the tumor was placed in the corresponding anatomical site in mice. This model showed that only carboplatinum and Salmonella A1-R were able to suppress CUP tumor growth.

Cancer of unknown primary (CUP) is a heterogeneous and resistant disease. CUP has a poor prognosis due to the lack of an effective standard first-line therapy. CUP usually involves apparent metastasis in patients where primary tumors cannot be found.^1^ CUP accounts for 3–5% of cancers, and its median survival time is only 3 months. CUPs are classified as differentiated adenocarcinomas (~60%), undifferentiated adenocarcinomas (30%), squamous cell carcinomas (5%), neuroendocrine tumors (~1%) and melanomas. The primary cancer site can eventually be diagnosed in 25% of CUP patients with improved imaging, immunohistochemistry, serum markers^2^ and genomic techniques.^3,4^ CUP patients have been treated with various chemotherapeutic drugs with variable success. CUP consists of heterogeneous malignancy groups, making personalized and precision therapy imperative. Toward this goal, we established the patient-derived orthotopic xenograft (PDOX) nude mouse model using the surgical orthotopic implantation (SOI) technique.^4,5^ Our PDOX model has many advantages over subcutaneous-transplant models.^6^ We previously reported that tumor-targeting *S. typhimurium* A1-R was effective against many types of PDOX models.^7,8^ The present study aimed to identify an effective therapy for a CUP patient using a PDOX model. In this report, we investigated the usefulness of a PDOX model for precisely identifying effective and ineffective chemotherapy. We also compared the efficacy of *S. typhimurium* A1-R with first-line chemotherapy using the CUP PDOX model.

The present study used 4- to 6-week-old, athymic *nu/nu* nude mice (AntiCancer, Inc., San Diego, CA, USA). All experimental protocols and data were collected as previously described.^4–8^ The patient CUP tumor was resected in a left neck lymph node in the Department of Surgery, University of California, Los Angeles (UCLA). The results from positron emission tomography (PET); bone scintigraphy; ear, nose and throat (ENT) evaluation; and endoscopy were all negative for tumors at other sites. Our pathological findings suggested that this tumor was metastatic, poorly differentiated neoplasm because it was epithelial membrane antigen-positive, periodic acid-Schiff (PAS)-negative, and P16-positive. Because the above findings were insufficient to diagnosis a primary tumor, this tumor was diagnosed as CUP.

**Fig. 1:**
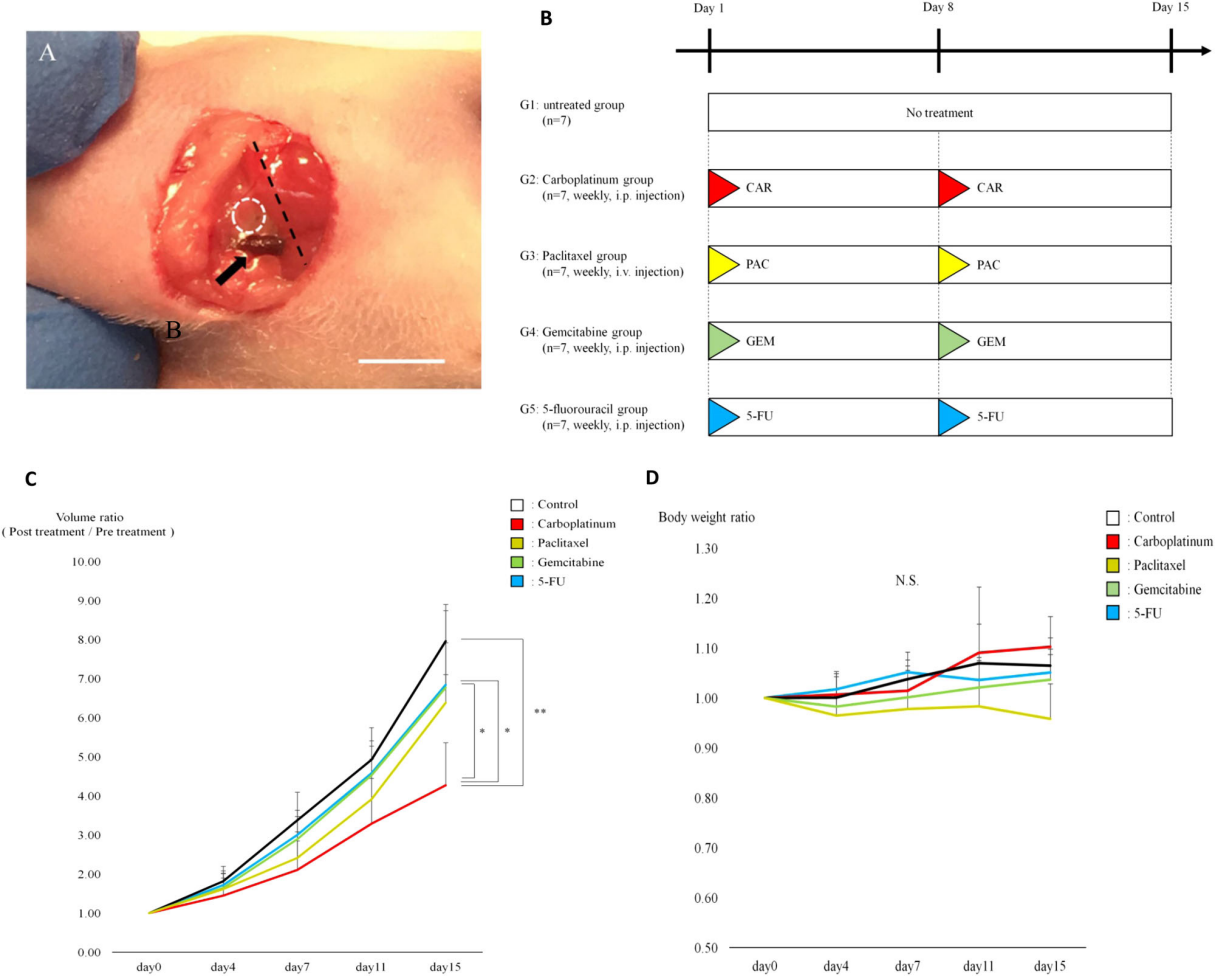
**a** Surgical orthotopic implantation (SOI). A 10-mm incision was made in the left neck of the nude mice. A small CUP tumor fragment (4 mm^3^) was implanted in the left supraclavicular fossa (white dotted circle) of the nude mice using SOI to establish the PDOX model. Black dotted line: left clavicle. Black arrow: left external jugular vein. Scale bar: 10 mm. **b** Treatment protocol. G1: untreated group; G2: carboplatinum (CAR) (30 mg/kg, i.p., weekly, 2 weeks); G3: paclitaxel (PAC) (20 mg/kg, i.v., weekly, 2 weeks); G4: gemcitabine (GEM) (100 mg/kg, i.p., weekly, 2 weeks); and G5: 5-fluorouracil (5-FU) (50 mg/kg, i.p., weekly, 2 weeks). Each group consisted of 7 mice. All mice were humanely killed on day 15. **c** Tumor growth. Line graphs show the relative tumor volume ratio (treatment day/day 0) throughout the treatment. CAR suppressed tumor growth significantly compared with the untreated group (*P* < 0.001). CAR was significantly more effective than GEM (*P* = 0.015) and 5-FU (*P* = 0.011). PAC (*P* = 0.224), GEM (*P* = 0.492) and 5-FU (*P* = 0.551) showed no significant efficacy compared with the untreated group. Error bars: ± SD. *P < 0.05, **P < 0.001. **d** Mouse body weight. Line graphs show the body weight ratios of each group (treatment day/day 0). No significant differences were found between the untreated and treated groups. Error bars: ± SD

The resected fresh tumor was brought to AntiCancer, Inc. from the UCLA Hospital, and the CUP PDOX tumor model was established in nude mice using the SOI technique to the left supraclavicular fossa (Fig. 1a and 3a, b). Detailed preparation, administration and imaging of *S. typhimurium* A1-R are described in the supplementary methods. CUP PDOX models were randomized into 5 groups when the tumor volumes reached 100 mm^3^, which are as folllows: G1: untreated; G2: carboplatinum (CAR); G3: paclitaxel (PAC); G4: gemcitabine (GEM); and G5: 5-fluorouracil (5-FU). This treatment scheme for the CUP PDOX model is shown in Fig. 1b. In addition, CUP PDOX models were also randomized into the following 5 groups to compare *S. typhimurium* A1-R with first-line chemotherapy when the tumor volume reached 100 mm^3^: G1: untreated control; G2: CAR; G3: GEM; G4: 5-FU; and G5: *S. typhimurium* A1-R. This treatment scheme for the CUP PDOX models is illustrated in Fig. 3b. Seven mice were used per group. All mice were humanely killed on day 15 after initiating treatment. Detailed statistical analyses are provided in the supplementary methods.

All animal studies were performed using an AntiCancer, Inc. Institutional Animal Care and Use Committee (IACUC) protocol specifically approved for this study and in accordance with the principles and procedures outlined in the National Institute of Health Guide for the Care and Use of Animals under Assurance Number A3873-1. All efforts were made to minimize the number of animals used and their suffering. For the patient study, informed consent was obtained from the patient under UCLA Institutional Review Board-approved protocol (IRB #10-001857) to perform a PDOX study.

We compared the efficacy of four drugs (CAR, GEM, PAC and 5-FU) on CUP PDOX tumor growth. Of the four drugs tested, we found that CAR significantly suppressed tumor growth more than the untreated group (P < 0.001) (Fig. 1c). In addition, CAR was significantly more effective than GEM (*P* = 0.015) and 5-FU (*P* = 0.011) (Fig. 1c). By contrast, PAC (*P* = 0.224), GEM (*P* = 0.492) and 5-FU (*P* = 0.551) showed no significant efficacy compared with the untreated group (Fig. 1c). The final tumor volume ratios (day 15/day 0) were as follows: untreated group (G1) (7.96 ± 0.78); CAR group (G2) (4.27 ± 1.1); PAC group (G3) (6.39 ± 0.72); GEM group (G4) (6.77 ± 2.1); and 5-FU group (G5) (6.84 ± 1.1) (Fig. 1c).

We also measured body weights of the mice pre-treated and post-treated with CAR, GEM, 5-FU, PAC, and GEM. Although the PAC group’s relative body weight decreased slightly, no significant difference was noted in body weight among the five groups (Fig. 1d).

**Fig. 2:**
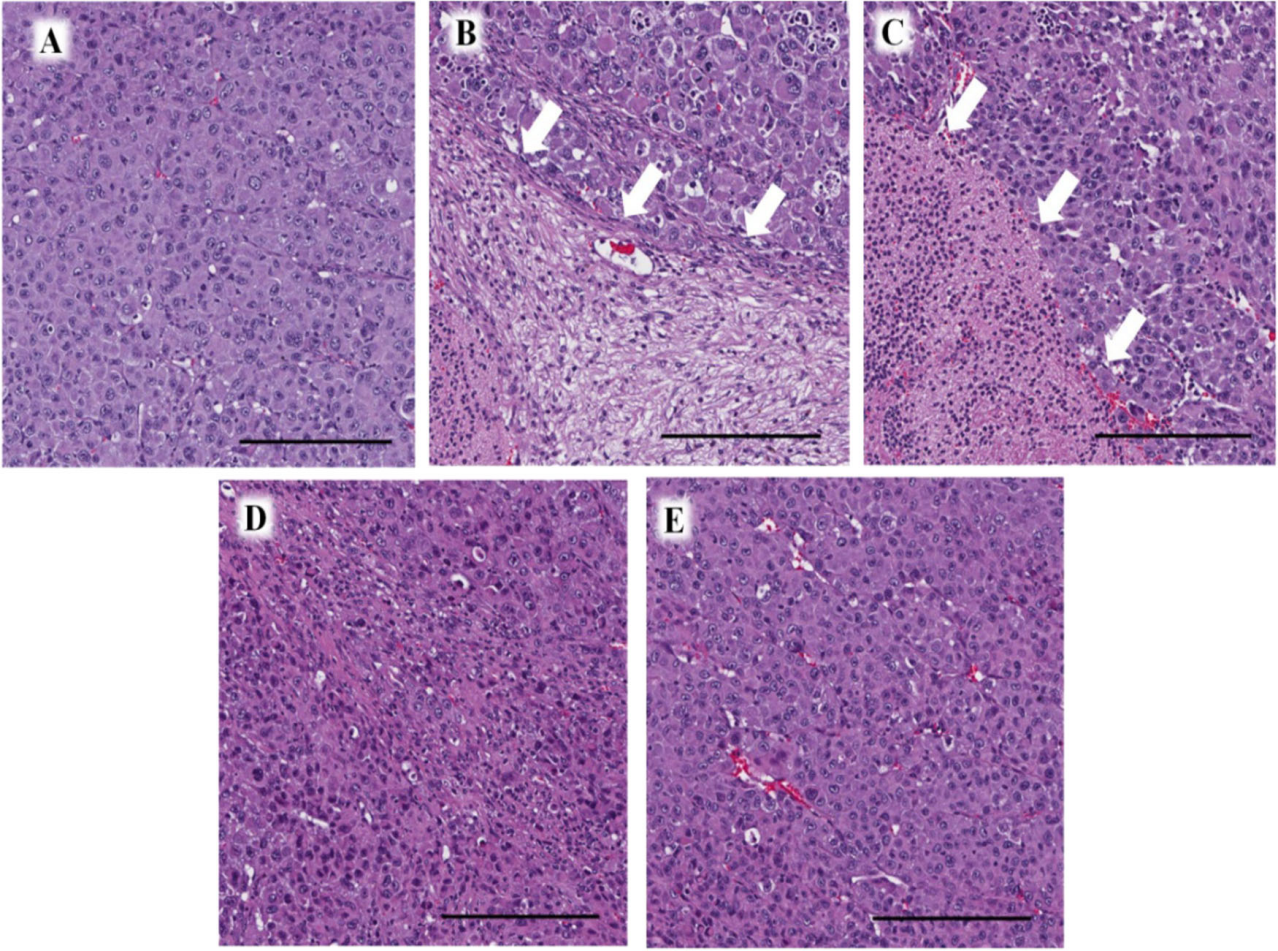
Tumor histology. **a** Hematoxylin and eosin (H&E) staining of the untreated CUP PDOX tumor. **b** H&E staining of the CAR-treated CUP PDOX tumor. Necrosis was observed (white arrows). **c** H&E staining of the PAC-treated CUP PDOX tumor. Necrosis was observed (white arrows). **d** H&E staining of the GEM-treated CUP PDOX tumor. **e** H&E staining of the 5-FU-treated CUP PDOX tumor. Scale bar: 200 µm

We carefully examined the histological sections of the treated and untreated groups. Necrosis due to chemotherapy was only found in the CAR- and PAC-treated groups (Fig. 2).

**Fig. 3:**
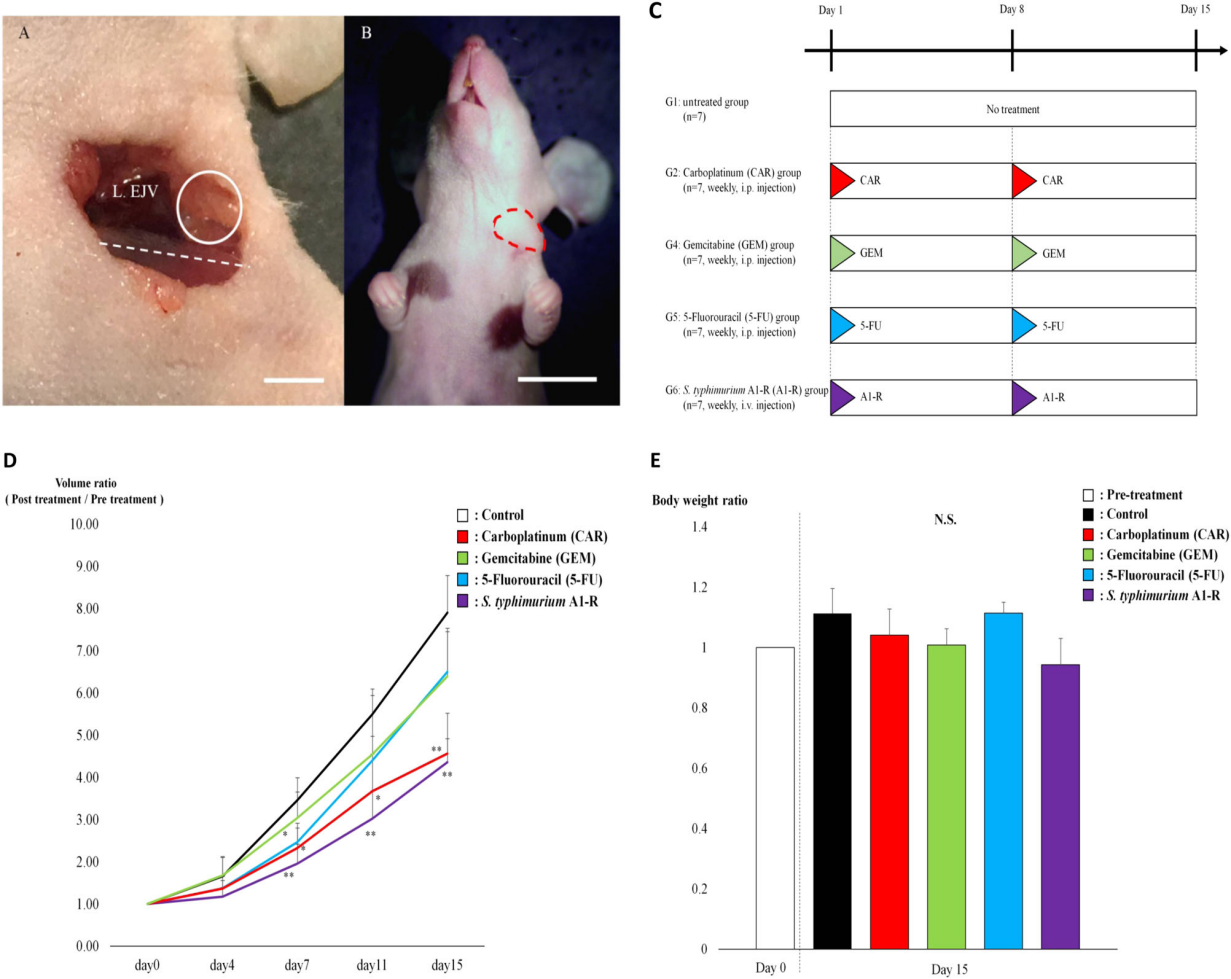
**a** Surgical orthotopic implantation (SOI). A 4-mm^2^ tumor fragment of the cancer of unknown primary (CUP) was implanted into the left supraclavicular fossa (white circle) of the nude mice using SOI to establish the PDOX model. White dotted line: left clavicle. Scale bar: 2 mm. **b** Detectable CUP PDOX tumors were established 10 days after SOI. The area surrounded by a red  dotted line shows the established tumor. Scale bar: 10 mm. **c** Treatment protocol. G1: untreated group; G2: carboplatinum (CAR) (30 mg/kg, i.p., weekly, 2 weeks); G3: gemcitabine (GEM) (100 mg/kg, i.p., weekly, 2 weeks); G4: 5-fluorouracil (5-FU) (50 mg/kg, i.p., weekly, 2 weeks); and G5: *Salmonella typhimurium* A1-R (*S. typhimurium* A1-R) (100 CFU/body, i.v., weekly, 2 weeks). Each group consisted of 7 mice. All mice were humanely killed on day 15. **d** Tumor volume ratio (Treatment day/Day 0). Line graphs show tumor volume ratios. *S. typhimurium* A1-R (*P* < 0.001) and CAR (*P* < 0.001) significantly inhibited tumor growth compared with the untreated group on day 15. GEM (*P* = 0.057), and 5-FU (*P* = 0.088) did not significantly suppress tumor growth compared with the untreated group on day 15. Error bars: ± SD. *P < 0.05, **P < 0.001. **e** Body weight per group. Bar graphs show the body weight ratios of each group (pre-treatment/post-treatment). No significant differences were found between any groups. Error bars: ± SD. L. EJV: left external jugular vein

The CUP PDOX model was also used to compare the efficacy between first-line therapy (CAR, GEM, 5-FU) and *S. typhimurium* A1-R (Fig. 3a–c). We found that CAR (*P* < 0.001) and *S. typhimurium* A1-R significantly (*P* < 0.001) inhibited tumor growth compared with the untreated control. No significant (*P* = 0.995) difference was observed in relative tumor volume between CAR and *S. typhimurium* A1-R. In addition, the efficacies of GEM (*P* = 0.057) and 5-FU (*P* = 0.088) were not statistically significant compared with the untreated control. The final tumor volume ratios (day 15/day 0) were as follows: untreated (G1) (7.90 ± 0.88); CAR (G2) (4.56 ± 0.96); GEM (G3) (6.39 ± 1.15); 5-FU (G4) (6.50 ± 0.95); and *S. typhimurium* A1-R (G5) (4.36 ± 0.56) (Fig. 3d). Mouse body weights were also measured before and after treatment with first-line therapy and *S. typhimurium* A1-R. Although the relative body weight of the *S. typhimurium* A1-R group slightly decreased, it was not significant (0.94 ± 0.09). Further, we found no significant differences in body weight between any groups (Fig. 3e).

**Fig. 4:**
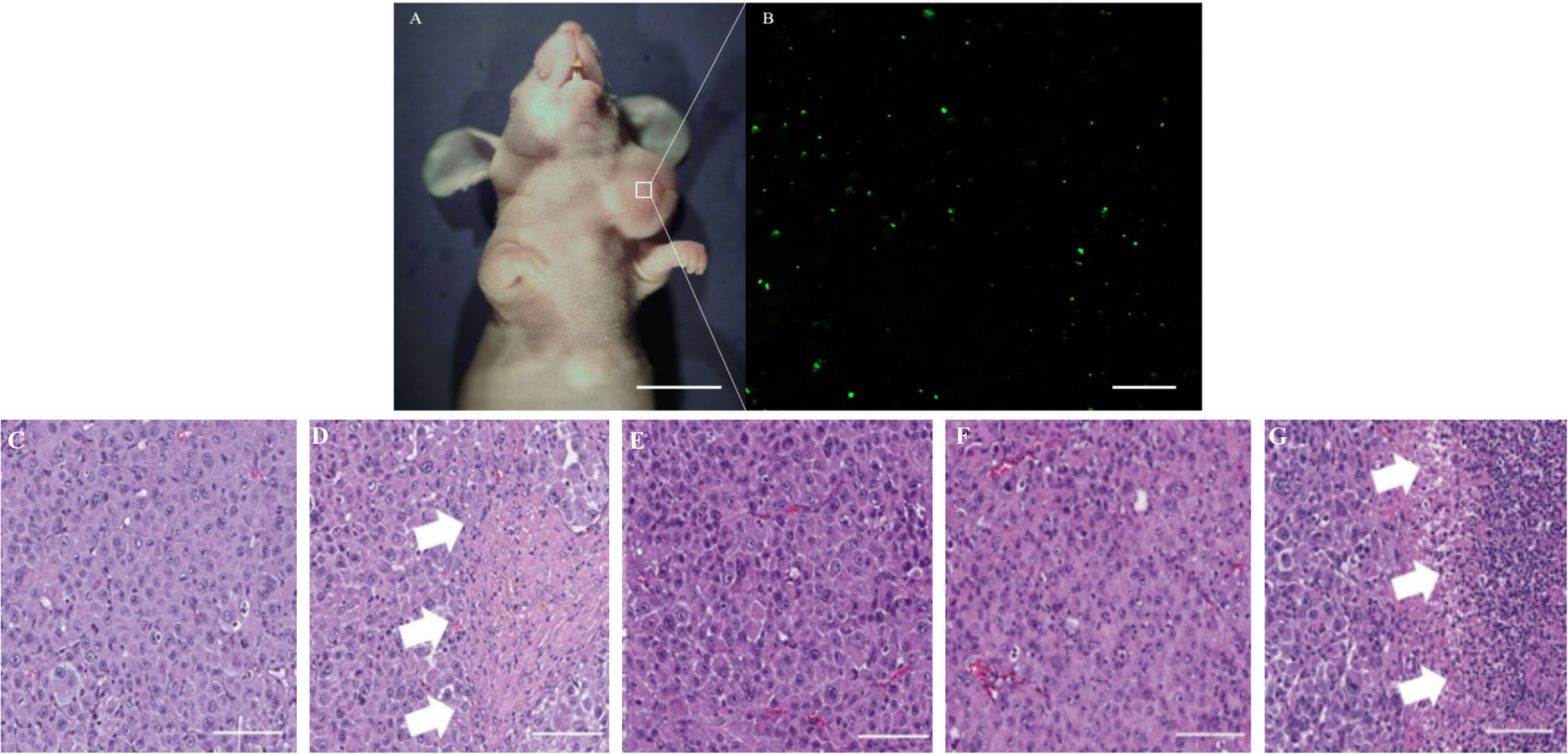
**a** Tumor treated with *S. typhimurium* A1-R GFP. Scale bar: 10 mm. **b** Accumulation of *S. typhimurium* A1-R GFP in the CUP PDOX tumor was demonstrated by confocal microscopy (FV1000). Scale bar: 50 μm. Tumor histology. **c** Hematoxylin and eosin (H&E) staining of the untreated CUP PDOX tumor. **d** H&E staining of the CAR-treated CUP PDOX tumor. Some necrosis was observed (white arrows). **e** H&E staining of the GEM-treated CUP PDOX tumor. **f** H&E staining of the 5-FU-treated CUP PDOX tumor. **g** H&E staining of the *S. typhimurium* A1-R-treated CUP PDOX tumor. Extensive necrosis was observed (white arrows). Scale bar: 100 µm

Distribution of *S. typhimurium* A1-R GFP (green fluorescent protein) tumor-targeting was confirmed by confocal imaging with the FV1000 (Olympus, Tokyo, Japan) (Fig. 4a, b).

We carefully examined the histological sections of the treated and untreated groups. Moderate necrosis was observed in the CAR group tumors (Fig. 4d). By contrast, the *S. typhimurium* A1-R-treated tumors showed massive necrosis (Fig. 4g).

The present study reports the first CUP PDOX model. A heterotopic subcutaneous xenograft CUP model was previously reported.^9^ The PDOX model placed the CUP tumor at the anatomic site of the mouse that corresponded with that of the patient.

A few studies show limited efficacy of targeted CUP therapies.^10^ Several chemotherapeutic combinations have limited efficacy on CUP.^11,12^ A few recent studies provided therapeutic approaches to target CUP.^13,14^ Other treatment regimens were also developed based on histologic type and gene expression.^15,16^ In addition, a recent study using next-generation sequencing identified a combinatorial strategy for CUP that targets tumor protein 53 (TP53)-associated genes, the mitogen-activated protein kinase (MAPK) pathway, phosphoinositide 3-kinase (PI3K) signaling, and cell-cycle-associated genes^17^ However, before novel therapeutic options become clinically viable, developing personalized, precision therapy is crucial.

In the present study, we initially tested four drugs with the CUP PDOX model: CAR, PAC, GEM, and 5-FU. These four drugs are first-line treatments for CUP per the National Comprehensive Cancer Network (NCCN) guidelines.^18^ Only CAR showed significant efficacy among the four drugs. These results suggested that a CAR-based regimen would be effective for this CUP patient and that the patient should not be treated with PAC, GEM or 5-FU. The present CUP PDOX study showed how the PDOX model can precisely distinguish between different therapies.

In addition, we demonstrated that *S. typhimurium* A1-R has more potential against the CUP PDOX  than first-line chemotherapy. *S. typhimurium* A1-R’s efficacy against CUP PDOX tumor growth is supported by the *S. typhimurium* A1-R GFP’s targeting into the tumor visualized by confocal fluorescence microscopy and by the severe necrosis in the tumor tissue (Fig. 4a, b).

These results have great impact since the CUP tumor grew at the corresponding site on the nude mice as it did in the patient. Thus, the tumor grew in the corresponding tumor microenvironment (TME) in both man and mouse.

On the basis of the recent findings, *S. typhimurium* A1-R could be a therapeutic option for cancers as it could inhibit or eliminate primary and metastatic tumors by destroying tumor blood vessels and/or elevating CD8+T-cell infiltration.^8,19^ In our previous studies, we reported that tumor-targeting *S. typhimurium* A1-R was highly effective in many PDOX models.^7,8,19^ On the basis of the previous studies on *S. typhimurium* A1-R’s efficacy in various PDOX cancer models and the present study on the CUP PDOX model, we suggest that tumor-targeting *S. typhimurium* A1-R has immense potential in curing recalcitrant cancers.^20^

In conclusion, our CUP PDOX model identified CAR as the only active drug among the 4 drugs that were tested. The CUP PDOX model has potential for precise, personalized treatment for CUP patients. We showed that although CAR was potentially effective as a first-line therapy, GEM and 5-FU were not. By contrast, *S. typhimurium* A1-R was significantly more effective than first-line therapy. Collectively, we provided evidence that the CUP PDOX could eliminate ineffective therapy and identified CAR as effective standard therapy and *S. typhimurium* A1-R as effective experimental therapy.

## Electronic supplementary material

Supplementary methods
