# Supplementary material for: Patient-derived orthotopic xenograft models for cancer of unknown primary precisely distinguish chemotherapy, and tumor-targeting S. typhimurium A1-R is superior to first-line chemotherapy
Source: Signal Transduct Target Ther. 2018 Apr 27;3:12. doi: 10.1038/s41392-018-0016-7 (PMC5923205; doi:10.1038/s41392-018-0016-7)
Supplement: Supplementary file 1 — Supplementary methods [file 41392_2018_16_MOESM1_ESM.pdf]

Supplementary information

Patient-derived orthotopic xenograft models of cancer of unknown primary precisely distinguish chemotherapy while tumor-targeting *S. typhimurium* A1-R is superior to first-line chemotherapy

Kentaro Miyake, Tasuku Kiyuna, Masuyo Miyake, Kei Kawaguchi, Sang Nam Yoon, Zhiying Zhang, Kentaro Igarashi, Sahar Razmjooei, Sintawat Wangsiricharoen, Takashi Murakami, Yunfeng Li, Scott D. Nelson, Tara A. Russell, Arun S. Singh, Yukihiko Hiroshima, Masashi Momiyama, Ryusei Matsuyama, Takashi Chishima, Shree Ram Singh, Itaru Endo, Fritz C. Eilber, and Robert M. Hoffman

Supplementary Methods

Mice

In the present study, 4–6 weeks old, Athymic *nu/nu* nude mice (AntiCancer Inc., San Diego, CA), were utilized. Animals were housed in a barrier facility on a high efficacy particulate arrestance (HEPA)-filtered rack under standard conditions of 12-hour light/dark cycles. The animals were fed an autoclaved laboratory rodent diet. All mouse surgical procedures and imaging were done with the animals anesthetized by subcutaneous injection of a ketamine mixture (0.02 ml solution of 20 mg/kg ketamine, 15.2 mg/kg xylazine, and 0.48 mg/kg acepromazine maleate). The reaction of animals during surgery was observed to ensure adequate depth of anesthesia. The animals were monitored daily and humanely sacrificed by CO_2 inhalation if they met the following humane endpoint criteria: severe tumor burden (more than 20 mm in diameter), prostration, significant body weight loss, difficulty breathing, rotational motion, and body temperature drop.

Establishment of the CUP PDOX model

The patient CUP tumor was resected in a left neck lymph node in the Department of Surgery, University of California, Los Angeles (UCLA). PET, bone scintigraphy, the ear, nose and throat (ENT) evaluation, and endoscopy were all negative for tumor at other sites. Pathological findings suggested that this tumor was a metastatic, poorly differentiated neoplasm due to the following: epithelial membrane antigen was positive, periodic acid-Schiff (PAS) was negative, and P16 was

positive. These findings were not sufficient to diagnosis a primary tumor and therefore this tumor was diagnosed as CUP. The resected fresh tumor was brought to AntiCancer Inc. from the UCLA Hospital and the tumor was established in nude mice using surgical orthotopic implantation (SOI) to the left supraclavicular fossa.

Preparation and administration of S. typhimurium A1-R

GFP-expressing *S. typhimurium* A1-R (AntiCancer Inc.,) was grown in LB medium (Fisher Sci., Hanover Park, IL, USA) and then diluted 1:10 in LB medium. Bacteria were harvested at late-log phase, washed twice with PBS, then diluted in phosphate-buffered saline (PBS) up to 5×10^8 colony-forming units (CFU)/ml. *S. typhimurium* A1-R (5×10^7 CFU) in 100 µl PBS were injected i.v. to each mouse.

Treatment protocol in the CUP PDOX model

The CUP PDOX models were randomly divided into the following 5 groups when the tumor volume reached 100 mm^3 according to UCLA Physicians' request; G1: untreated group; G2: carboplatinum (CAR) (30 mg/kg, i.p., weekly, 2 weeks); G3: paclitaxel (PAC) (20 mg/kg, i.v., weekly, 2 weeks); G4: gemcitabine (GEM) (100 mg/kg, i.p., weekly, 2 weeks); G5: 5-fluorouracil (5-FU) (50 mg/kg, i.p., weekly, 2 weeks) (Fig. 1B). In the CUP PDOX model to compare *S. typhimurium* A1-R with first-line chemotherapy, the CUP PDOX models were also divided randomly into 5 groups after the tumor volume reached 100 mm^3: G1: untreated group; G2: CAR (30 mg/kg, i.p., weekly, 2 weeks); G3: GEM (100 mg/kg, i.p., weekly, 2 weeks); G4: 5-FU (50 mg/kg, i.p., weekly, 2 weeks); G5: *S. typhimurium* A1-R (100 CFU / body, i.v., weekly, 2 weeks) (Fig. 3B). Each group comprised of 7 mice. Dosages of the above drugs were determined from the literature.[8] Tumor volume and body weight were measured twice a week using the following formula: tumor volume (mm^3) = length (mm) x width (mm) x width (mm) x 1/2. All mouse models were sacrificed on day 15.

2.5. Visualization of tumor-targeting by *S. typhimurium* A1-R

Fluorescence images were obtained of *S. typhimurium* A1-R-GFP targeting the CUP tumor with the FV1000 confocal microscope with 20×/0.50 UPlan FLN and 40×/1.3 oil Olympus UPlan FLN objectives (Olympus, Tokyo, Japan).

2.6. Histological examination

10% formalin fixed, paraffin embedded tumor tissue sections (5 μm) were deparaffinized in xylene and rehydrated in an ethanol series. Hematoxylin and eosin (H&E) staining was performed according to standard protocol**.** Histological examination was observed by a BHS system microscope (Olympus Corp., Tokyo, Japan).

2.7. Statistical analysis

All statistical analyses were performed by Statistical Package for the Social Sciences for Windows software version 22.0 (IBM Corp., Armonk, NY, USA). Bartlett's test was used to verify the homogeneity of variances among the 5 groups. Significant differences for comparisons of 5 groups were determined using one-way ANOVA followed by Tukey post hoc pairwise tests. Line graphs showed average and error bar expressed ± standard deviation. A probability value of $P < 0.05$ was defined as statistical significance.
